# Novel Method for the Rapid Establishment of Antibiotic Susceptibility Profiles in Bacterial Strains Linked to Musculoskeletal Infections Using Scattered Light Integrated Collector Technology

**DOI:** 10.3390/ijms26041553

**Published:** 2025-02-12

**Authors:** Damien Bertheloot, Vincent B. Nessler, Elio Assaf, Cosmea F. Amerschläger, Kani Ali, Robert Ossendorff, Max Jaenisch, Andreas C. Strauss, Christof Burger, Phillip J. Walmsley, Gunnar T. Hischebeth, Dieter C. Wirtz, Robert J. H. Hammond, Frank A. Schildberg

**Affiliations:** 1 Department of Orthopedics and Trauma Surgery, University Hospital Bonn, 53127 Bonn, Germany; 2 School of Medicine, University of St Andrews, St Andrews KY16 9TF, UK; 3 Institute for Medical Microbiology, Immunology and Parasitology, University Hospital Bonn, 53127 Bonn, Germany

**Keywords:** bacteria, antibiogram, resistance, periprosthetic joint infections

## Abstract

Bacterial antibiotic resistance is an important challenge that the healthcare system is continually battling and a major problem in the treatment of musculoskeletal infections such as periprosthetic joint infections. Current methods to identify infectious microbes and define susceptibility to antibiotics require two to ten days from isolation to the establishment of an antibiogram. This slow process limits advances in antimicrobial drug discovery and, in the clinical context, delays the delivery of targeted treatments, with potentially devastating outcomes for patients. With this in mind, we strived to establish a quicker and more sensitive method to deliver antibiotic susceptibility profiles of clinically relevant microbes using Scattered Light Integrated Collector (SLIC) technology. We established antibiotic panels to obtain an approximate identification of a wide variety of microbes linked to periprosthetic joint infections and determine their susceptibility to antibiotics. We challenged microbes isolated from patients with our tailored antibiotic panels and found that SLIC detects perturbations in bacterial growth accurately and reproducibly within minutes of culture. Indeed, we could show that SLIC can be used to measure the dose-dependent inhibitory or bacteriolytic activity of broad classes of antibiotics. Our panel design enabled us to establish a profile similar to an antibiogram for the tested bacteria within 90 min. Our method can provide information on the class of bacteria tested and potential treatment avenues in parallel. Our proof-of-principle experiments using isolated clinical strains of bacteria demonstrate that SLIC, together with our specifically designed antibiotic panels, could be used to rapidly provide information on the identity of an infecting microbe, such as those associated with periprosthetic joint infections, and guide physicians to prescribe targeted antibiotic treatment early-on. The constant emergence of resistant strains of bacteria pushes the pharmaceutical industry to develop further effective drugs. Our optimized method could significantly accelerate this work by characterizing the efficacy of new classes of compounds against bacterial viability within minutes, a timeframe far shorter than the current standards.

## 1. Introduction

Total hip or knee replacement surgeries are among the 10 most practiced surgeries in Europe. Considering the aging of our population, the total number of surgeries is predicted to increase further, reaching over 530M surgeries in Germany by 2040 [1]. One major complication of these surgeries is the development of an infection, also referred to as periprosthetic joint infection (PJI), causing 22% of all revision surgeries [2,3], with over 20% at risk of mortality within 5 years [4,5]. Hence, it is essential to diagnose PJI as quickly as possible to increase the patient’s chances of survival. Among other criteria, several positive periprosthetic cultures are the main criterion for the diagnosis of PJI [6,7]. The primary type of microorganism causing PJI are bacteria derived from the skin flora (penetrating through the wound) or from diverse mucosa (e.g., gastrointestinal or respiratory) from which the route of penetrance into the joint tissue is unclear, although most likely hematogenous [8,9]. With current technologies, the detection of infection can take several days, especially for gram-positive bacteria [10], during which time patients are treated empirically following hospital-specific protocols, with variable success [11].

Finding a precise susceptibility profile of the infecting microbe usually first requires its culture (on solid or liquid media), identification (using various techniques from simple staining, to PCR or MALDI-TOF technologies, for more advanced facilities), and finally susceptibility testing (e.g., disk diffusion assay or more automated systems). Although new technologies are emerging to accelerate this process, these are not yet widespread in part because of their higher running costs [12,13,14,15]. The ever-developing issue of antimicrobial resistance (AMR) further amplifies the strain on clinics to effectively treat infections. Indeed, when ineffective, the use of empirical antibiosis increases the risk of the development of resistance in the treated microbe. At the same time, the increased prevalence of resistant bacteria in our environment further amplifies the failure potential of empirical treatments. There is therefore an urgent need for the development of technologies that combine speed, ease of use, and low running costs.

The Scattered Light Integrated Collector (SLIC) detects the scattering of laser light as a direct result of the presence of particulates in a solution. The technology is able to measure bacterial growth in real-time and with extreme sensitivity, allowing the detection of microorganism concentrations as low as 10^2^ cfu mL^−1^ [16]. Here, we aimed to establish a protocol that could deliver antibiotic susceptibility profiles of clinically relevant microbes within minutes. For this, we used SLIC technology combined with tailored antibiotic panels relevant to the field of orthopedics. We show that the SLIC detects the strain-specific and dose-dependent activity of a wide range of antibiotics in real time. We use the strain-selectivity of antibiotics like vancomycin to provide basic information on the type of bacteria tested (e.g., gram-positive vs. gram-negative). We determine the susceptibility profile of a variety of patient-derived strains to clinically relevant antibiotics within 90 min. Our data are the first proof-of-principle for the use of SLIC and dedicated antibiotics panels to rapidly diagnose an infection and deliver the susceptibility profile of the responsible microorganism in parallel. When adapted for direct use in peri-operative samples, this method could represent a cheap and easy-to-use solution to reduce the duration of, or even avoid the need for, empirical antibiosis.

## 2. Results

### 2.1. SLIC Detects Bacterial Growth and Dose-Dependent Antibiotic Activity and Selectivity

**Figure 1:**
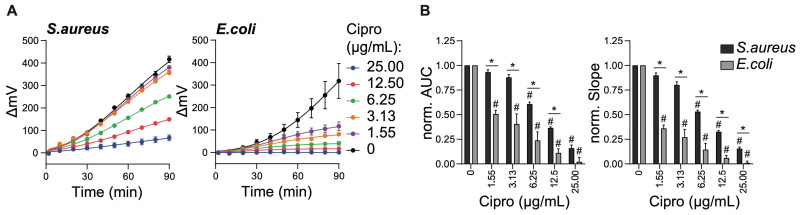
Antibiotic activity against bacterial lab strains measured using SLIC. Lab strains of *S. aureus* (ATCC 29213) and *E. coli* (WK6) were grown at 30 °C for 16 h. Following a 100-fold dilution and treatment with the indicated doses of Ciprofloxacin (Cipro), cell growth was monitored in real-time for 90 min inside the SLIC chamber set at 37 °C. (**A**) Normalized light refraction (ΔmV) as measured over time for the growth of *S. aureus* (left panel) or *E. coli* (right panel). Data points represent the average of the 60 measurements (1/s) taken every 10 min. (**B**) Normalized AUC (left panel) and normalized slope (right panel) of the refraction curves as shown in (**A**). Data are expressed as average ± SEM of 3 independent experiments. * *p* ≤ 0.05 comparing *S. aureus* with *E. coli* data and # *p* ≤ 0.05 comparing each antibiotic dose to its corresponding no treatment control (0 µg/mL) within each bacterial strain, calculated by multiple comparison Tukey test.

SLIC technology enables us to quantify the kinetics of bacterial growth with a great sensitivity superior to spectrophotometry [16]. Using SLIC, we first measured the growth of lab strains of *S. aureus* (ATCC 29213) or *E. coli* (WK6) when cultured in the presence of a titration of ciprofloxacin (0–25 µg/mL). As expected, bacteria grown in the absence of antibiotics showed a rapid growth along the 90 min recording time (Figure 1A). More importantly, the growth of both bacterial strains was dose-dependently inhibited by treatment with ciprofloxacin. Calculating the area under the curve (AUC), normalized to the uninhibited growth for each strain, allows us to better compare the activity of ciprofloxacin against bacterial growth and run statistical analysis. While the lowest ciprofloxacin dose we used (1.55 µg/mL) inhibited 50% of *E. coli* growth, it took four times more antibiotics (6.25 µg/mL) to significantly lower the growth of *S. aureus* (Figure 1B, left panel). Calculating the normalized average slope from the bacterial growth curves also revealed these differences in the susceptibility of *S. aureus* and *E. coli* to ciprofloxacin treatment (Figure 1B, right panel).

**Figure 2:**
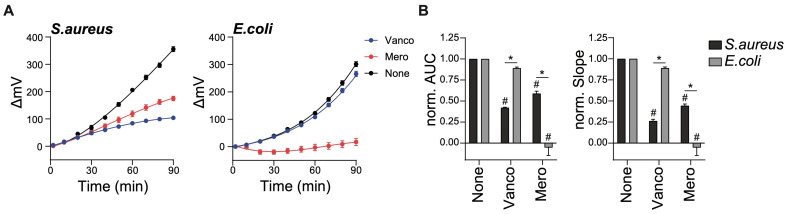
SLIC differentiates antibiotic susceptibility profiles of distinct bacterial strains. Lab strains of *S. aureus* (ATCC 29213) and *E. coli* (WK6) were grown at 30 °C for 16 h. Following a 100-fold dilution and treatment with either Vancomycin (3.13 µg/mL) or Meropenem (1.55 µg/mL), cell growth was monitored in real-time for 90 min inside the SLIC chamber set at 37 °C. (**A**) Normalized light refraction (ΔmV) as measured over time for the growth of *S. aureus* (left panel) or *E. coli* (right panel). Data points represent the average of the 60 measurements (1/s) taken every 10 min. (**B**) Normalized AUC (left panel) and normalized slope (right panel) of the refraction curves as shown in (**A**). Data are expressed as average ± SEM of 3 independent experiments. * *p* ≤ 0.05 comparing *S. aureus* with *E. coli* data and # *p* ≤ 0.05 comparing each antibiotic dose to its corresponding no treatment control (0 µg/mL) within each bacterial strain, calculated by multiple comparison Tukey test.

While ciprofloxacin is a very broad antibiotic, we next asked whether SLIC could be used to differentiate the susceptibility of *S. aureus* and *E. coli* to more specific antibiotics. For this, we measured the growth of *S. aureus* or *E. coli* in the presence of the gram-positive-specific antibiotic vancomycin. As expected, vancomycin inhibited over 50% of *S. aureus* growth but had no effect on *E. coli* growth (Figure 2A). On the other hand, meropenem is known to be highly potent against *E. coli* and somewhat weaker against *S. aureus* (MIC50 of 0.016 mg/mL or 0.125 mg/mL, respectively) [17]. When used as treatment against the growth of *S. aureus* and *E. coli*, we found that meropenem abrogated the growth of *E. coli* at all doses tested (Figure 2A, right panel). Yet, meropenem only partially inhibited the growth of *S. aureus* (Figure 2A, left panel). The normalized AUC and slope from the growth curves further highlight these findings (Figure 2B).

### 2.2. Optimization of Antibiotic Panels for Testing of Bacteria Susceptibility Profiles Using SLIC

**Figure 3:**
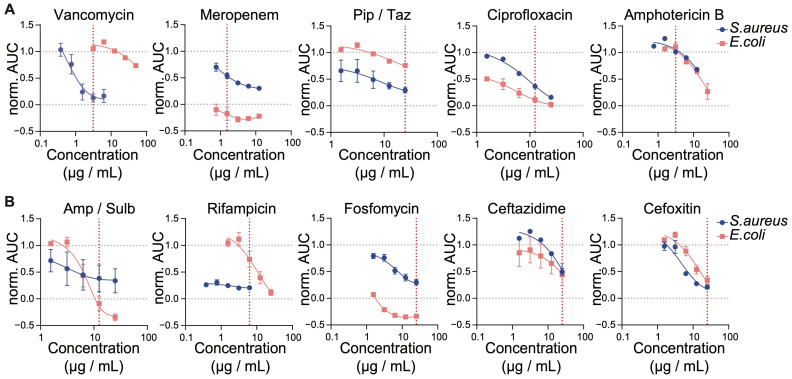
Establishment of MICs for all panel antibiotics. Lab strains of *S. aureus* (ATCC 29213) and *E. coli* (WK6) were grown at 30 °C for 16 h. Following a 100-fold dilution and treatment with the indicated titration of antibiotics, cell growth was monitored in real-time for 90 min inside the SLIC chamber set at 37 °C. Graphs represent the normalized AUC for each growth curve against the corresponding antibiotic concentration for antibiotics of Panel 1 (**A**) or Panel 2 (**B**). For combinations, concentration of piperacillin and ampicillin are displayed. Red dotted lines represent the selected MIC (see also Table 1). Data are expressed as average ± SEM of 3 independent experiments.

With our promising data demonstrating that SLIC can be used to measure the dose-dependent and strain-specific inhibitory activity of antibiotics, we next established two sets of five antibiotics. To provide a rough identification of an unknown infecting microbe, we selected antibiotics known for their activity against defined types of bacteria (e.g., vancomycin). We also chose clinically relevant antibiotics, especially in the field of orthopedic medicine (Table 1). The first panel of antibiotics included vancomycin, meropenem, the combination of piperacillin and tazobactam, ciprofloxacin, and amphotericin B. The second panel included the combination of ampicillin and sulbactam, rifampicin, fosfomycin, ceftazidime, and cefoxitin. As a first step, we titrated these antibiotics against the growth of *S. aureus* and *E. coli*, two common examples of either gram-positive or gram-negative bacteria, respectively (Figure 3).

Based on their inhibitory effect as measured with SLIC, we identified for each antibiotic a minimum inhibitory concentration (MIC) to be used in the following experiments (Figure 3, red dotted lines, Table 1). As seen in our previous experiments (Figure 2), vancomycin specifically targets gram-positive bacteria. We thus selected the dose that abrogated the growth of *S. aureus* while having no effect on *E. coli* (3.13 µg/mL, Figure 3A and Figure S1A). In the case of meropenem, we chose a dose inducing a 50% decrease in the growth of *S. aureus* while fully inhibiting the growth of *E. coli* (0.76 µg/mL, Figure 3A and Figure S1B). In our hands, the combination of piperacillin and tazobactam had a moderate effect on the growth of *E. coli* and *S. aureus*. We thus selected the highest dose tested for the following experiments (25 µg/mL and 3.13 µg/mL, respectively, Figure 3A and Figure S1C). Expecting ciprofloxacin to inhibit the growth of both *S. aureus* and *E. coli*, we chose the lowest dose enabling an almost full abrogation of *E. coli* growth (12.5 µg/mL, Figure 1A and Figure 3A). Amphotericin B has been developed to target the growth of fungi. Therefore, we chose the highest dose having no antagonistic effect on either *S. aureus* or *E. coli* to eliminate potential off-target toxicity effects (3.13 µg/mL, Figure 3A and Figure S1D). The combination of ampicillin and sulbactam is recommended to overcome the ever-developing resistance of bacteria to ampicillin, while allowing a de-escalation of the antibiotic therapy in terms of side-effects (e.g., instead of using meropenem). Here, we chose the lowest dose reaching full inhibition of *E. coli* and partial (50%) inhibition of *S. aureus* growth (12.5 µg/mL and 6.25 µg/mL, respectively, Figure 3B and Figure S2A). As two very relevant antibiotics in the context of orthopedic medicine, rifampicin and fosfomycin are broad-spectrum antibiotics with activity against biofilm-forming bacteria. In our experiments, rifampicin showed stronger activity against *S. aureus*, while fosfomycin was a stronger inhibitor of *E. coli*. Hence, MICs for rifampicin and fosfomycin were chosen to enable the differentiation of these two bacterial species (i.e., 6.25 µg/mL and 25 µg/mL, respectively, Figure 3B and Figure S2B,C). Ceftazidime is another broad-spectrum antibiotic used in the context of bone and joint infection (particularly against gram-negative bacteria) and a good alternative to the piperacillin/tazobactam combination. In our experiments, ceftazidime had similar activity against *S. aureus* or *E. coli*. We thus selected the dose with the best effect (25 µg/mL, Figure 3B and Figure S2D). Finally, cefoxitin is a broad-spectrum antibiotic often used for screening of MRSA. In our hands, cefoxitin equally inhibited the growth of *S. aureus* and *E. coli*. Hence, we selected the dose with the best antimicrobial activity (25 µg/mL, Figure 3B and Figure S2E) as the MIC.

**Figure 4:**
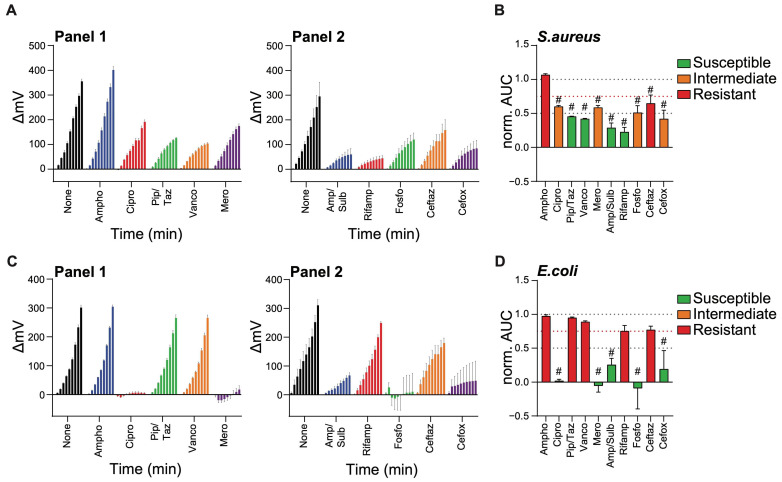
Antibiotic panels allow for the establishment of antibiogram-like profiles. Lab strains of *S. aureus* (ATCC 29213) and *E. coli* (WK6) were grown at 30 °C for 16 h. Following a 100-fold dilution and treatment with indicated antibiotics at the pre-established MIC (see Table 1), cell growth was monitored in real-time for 90 min inside the SLIC chamber set at 37 °C. (**A**,**C**) Data represents the normalized light refraction (ΔmV) as measured over time for the growth of *S. aureus* (**A**) or *E. coli* (**C**). Each data point represents the average of the 60 measurements (1/s) taken every 10 min. (**B**,**D**) Normalized AUC of the refraction growth curves as shown in (**A**) or (**C**), respectively. Bacteria were considered susceptible (green), intermediate (orange), or resistant (red) to indicated antibiotics if norm. AUCs are below 0.5, between 0.5 and 0.75, or above 0.75, respectively (see dotted lines). Data are expressed as average ± SEM of 3 independent experiments. # *p* ≤ 0.05 comparing each antibiotic treatment to the non-inhibited control (i.e., norm. AUC = 1), calculated by multiple comparison Tukey test.

We next tested both antibiotic panels with optimized MICs against the growth of *S. aureus* and *E. coli* lab strains (Figure 4). Here, we considered growth reduction of more than 50% (i.e., norm. AUC + SEM < 0.5) as susceptibility to the corresponding antibiotic (green). Growth inhibition of over 25% but less than 50% (i.e., 0.5 < norm. AUC +SEM < 0.75) was considered as intermediate susceptibility (orange). Finally, bacteria were considered resistant to an antibiotic when it caused less than 25% inhibition in growth (i.e., norm. AUC > 0.75, red).

As expected, *S. aureus* was susceptible to piperacillin/tazobactam, vancomycin, ampicillin/sulbactam, and rifampicin (Figure 4A,B). Ciprofloxacin, meropenem, Fosfomycin, and cefoxitin were somewhat less efficient at inhibiting the growth of *S. aureus* (i.e., less than 50%), although still significantly so. While ceftazidime significantly inhibited *S. aureus* growth, it did not significantly reach a degree of inhibition over 25%. Hence, here we considered *S. aureus* resistant to ceftazidime. As expected, amphotericin had no effect on the growth of *S. aureus*. Similarly, *E. coli* was resistant to amphotericin, as well as to piperacillin/tazobactam, vancomycin, rifampicin, and ceftazidime (Figure 4C,D). In contrast, *E. coli* was strongly susceptible to ciprofloxacin, ampicillin/sulbactam, and cefoxitin. We could even detect the cell lysing activity of meropenem and fosfomycin against *E. coli* (i.e., norm. AUC < 0).

### 2.3. SLIC Detects Differences in Susceptibility Profiles of Clinically Relevant Bacterial Strains

Next, we strived to test our antibiotic panels on clinically relevant bacterial strains. For this, we isolated bacteria from either synovial fluids or joint tissues (capsula or periprosthetic membrane) obtained peri-operatively from eight separate patients presenting with suspected PJI. First, we extracted the genomic DNA of the isolated bacteria and ran PCRs targeting the 16S ribosomal sequence (Figure S3A). We then identified the isolated strains by running SANGER sequencing on the purified PCR products. Sequences were compared to the NCBI 16S ribosomal sequences library (BLASTn, Figure S3B). Some sequences allowed us to identify clearly the bacterial strain isolated (e.g., *S. aureus* or *E. faecalis*). Other sequences had a 100% identity with more than one strain. Nonetheless, bacterial strains identified by MALDI-TOF—a routine microbiology diagnostic run as part of the patient’s medical care—corresponded to one of the top three strains identified by our SANGER sequencing (Figure S3), helping us to determine the exact nature of the bacterial strain we tested.

**Figure 5:**
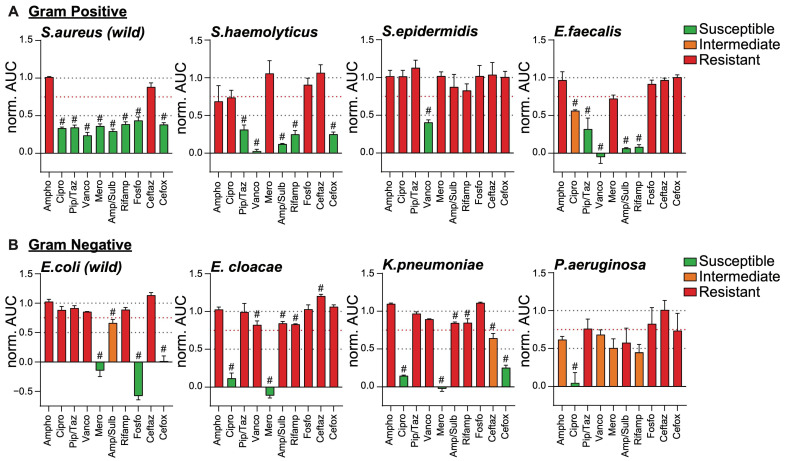
Antibiogram-like profiles of clinically relevant bacterial strains. Indicated bacterial strains were isolated from infected patient samples (peri-operative synovial fluid or tissue) and identified using PCR and SANGER sequencing of S16 ribosomal sequence (see Figure S3). Bacteria were grown at 30 °C for 16 h. Following a 100-fold dilution and treatment with indicated antibiotics at the pre-established MIC (see Table 1), cell growth was monitored in real-time for 90 min inside the SLIC chamber set at 37 °C. Data represents the Normalized AUC of the refraction growth curves (see also Figures S4 and S5). (**A**) Gram-positive and (**B**) gram-negative bacteria were considered susceptible (green), intermediate (orange), or resistant (red) to indicated antibiotics if norm. AUCs are below 0.5, between 0.5 and 0.75, or above 0.75, respectively (see dotted lines). Data are expressed as average ± SEM of 3 independent experiments. # *p* ≤ 0.05 comparing each antibiotic treatment to the non-inhibited control (i.e., norm. AUC = 1), calculated by multiple comparison Tukey test.

*S. aureus* is among the main microorganisms responsible for PJI [13,18,19,20]. Similarly, to the response of the lab strain, the patient-derived wild strain of *S. aureus* was susceptible to most antibiotics we tested, except for amphotericin B and ceftazidime, against which *S. aureus* was resistant (Figure 5A, left panel and Figure S4A). The *E. coli* wild strain we tested responded similarly to the lab strain to the antibiotic panels except for a surprisingly strong resistance to ciprofloxacin (Figure 5B, left panel, and Figure S5A). *S. haemolyticus* is a great example of a skin flora-originating microbe often found to cause PJI. In our experiments, *S. haemolyticus* was susceptible to piperacillin/tazobactam, vancomycin, ampicillin/sulbactam, rifampicin, and cefoxitin (Figure 5A, middle left panel and Figure S4B). As expected, it was resistant to amphotericin B. More unexpectedly, we found *S. haemolyticus* was resistant to ciprofloxacin, meropenem, fosfomycin, and ceftazidime, although resistance of certain *S. haemolyticus* strains to these drugs has been reported [21]. Another example of skin flora-originating bacteria causing PJI, *S. epidermidis* surprisingly resisted all antibiotics we tested, except for vancomycin (Figure 5A, middle right panel, and Figure S4C). *E. faecalis* is a good example of a gut mucosal bacteria often found in PJI. It responded similarly to *S. haemolyticus* against our antibiotics panels, except for its intermediate response to ciprofloxacin and its expected resistance to cefoxitin [22] (Figure 5A, middle right panel and Figure S4D). On the gram-negative side of mucosa-originating bacteria, *E. cloacae* showed strong resistance to most antibiotics tested (Figure 5B, middle left panel, and Figure S5B). Only ciprofloxacin and meropenem efficiently inhibited the growth of this *E. cloacae* strain. Interestingly, beyond inhibition, meropenem showed lysing activity (i.e., negative normalized AUC). The lung mucosal bacteria *K. pneumoniae* had a very similar antibiotic susceptibility profile to that of *E. cloacae*, with ciprofloxacin and meropenem having the best inhibitory activity. Yet, *K. pneumoniae* was also susceptible to cefoxitin and was partially inhibited by ceftazidime (Figure 5B, middle right panel and Figure S5C). *P. aeruginosa* is a gram-negative bacteria often diagnosed as the cause of PJI [18,20] and unfortunately often difficult to treat. The strain we isolated from the infected joint of a patient was resistant or only intermediately responded to all antibiotics but ciprofloxacin, potentially reflecting the challenges faced by the clinicians to find a treatment strategy (Figure 5B, right panel, and Figure S5D).

Together, our data demonstrated that SLIC is a sensitive readout of bacterial growth that has the potential to accelerate the diagnosis of infection and establish an antibiotic susceptibility profile in time to avoid, or at least limit, empirical treatment. SLIC could thus represent a significant breakthrough for providing patients with targeted—essentially personalized—treatment options.

## 3. Discussion

The discovery of antibiotics revolutionized the field of medicine and saved countless lives. Yet, partly because of overuse and misuse, the increased occurrence of (multi-)antimicrobial resistance in our environment presents the next challenge for our health systems and societies [23]. The accumulation of resistant microbial strains in our surroundings [24] has led to an increase in infections that defeat common treatment strategies. In the field of orthopedic surgery, the occurrence of periprosthetic joint infections (PJIs) has remained stable at around 2% of the total number of arthroplasties. However, the aging of our population drives up the number of arthroplasties (TKA and THA) [1], thus also increasing the total number of PJI cases. Failure to treat these infections rapidly can lead to the progressive expansion of infections, which can lead to bacteremia and sepsis. In fact, the 5-year mortality rate of patients with PJI exceeds 20% [4,5]. It is crucial to rapidly identify the causative microbial agent as well as its antimicrobial susceptibility profile to enable targeted treatment. Unfortunately, current microbiology diagnostic procedures may take several days to identify the causative microbe, followed by at least several hours to run susceptibility tests adapted to the specific strain [10,25]. In the meantime, patients are treated empirically, often with limited success [11].

We propose the use of SLIC technology combined with clinically relevant antibiotic panels as a fast add-on to the current microbiology diagnostic protocols. We could show that SLIC delivers fast dose-dependent and strain-specific susceptibility profiles of clinically relevant bacterial strains within minutes. Our tailored panel of antibiotics covered all bacterial strains isolated from the joint fluid or tissue of orthopedic patients, and provided several therapeutic options including the possibility for treatment de-escalation. Interestingly, we could efficiently detect the resistance of strains to antibiotics that were predicted to curtail the microbe’s growth. One must also mention the extreme affordability of this technology, as it only requires growth medium, common spectrophotometry cuvettes, and the selected antibiotics, on top of the dedicated instrument.

Although SLIC shows clear benefits in terms of sensitivity and speed, when compared to methods like disc diffusion, our current method still requires the culture of the infecting microbe before detection of its susceptibility profile using SLIC. The next step will be to optimize our protocol using primary samples thus avoiding the need for previous culture and isolation of the infecting microbe. This would dramatically shorten the time for detection of the infection and, more importantly, avoid the need for an empirical treatment or at least minimize its duration. The testing of primary samples will also require the inclusion of larger cohorts of patients infected with a wider variety of microbes to define more precisely the resolution of our method and demonstrate its efficacy across the large variety of infectious microbes found in the environment.

SLIC technology is in its infancy, and current instruments do not allow the necessary throughput for routine use in diagnostics. Despite this, our results represent a true proof of concept, motivating further development in hardware design. While our current setup only tests one dose for each antibiotic (based on our MIC determination), a significant increase in throughput would also make it possible to test several doses around our estimated MIC. This would provide more resolution in the susceptibility profiles of the tested bacteria. Furthermore, the present antibiotic panels were designed based either on their specificity to defined types of bacteria (e.g., vancomycin was used to determine the gram identity) or because of their relevance to treatment strategies in the field of orthopedic medicine. These panels could of course be adapted or expanded to include antibiotics used frequently to treat other tissue infections. Our present analysis was limited by the hardware design, which currently allows us to test five antibiotics in parallel with one uninhibited control. The technology has the capacity for this to be successfully increased to test more than five with a larger cell design. Aside from quickly providing clinically crucial information on how to treat the tested infection, the susceptibility profile generated by the setup presented here can give a rough estimation of the type of bacteria being tested (e.g., gram-negative vs gram-positive). It is currently no match for the precision provided by MALDI-TOF or sequencing. We consider our method as an important add-on to the current diagnostic procedures rather than a replacement. Considering the large variety of bacterial species and their ever-varying susceptibility to antibiotics, even provided with an instrument capable of higher throughput, it is rather unlikely that susceptibility profiles will ever generate enough information to provide a precise identification. Infections with mixed bacterial populations represent another level of complexity. Although this would require further testing, we predict our method would be capable of differentiating strains with different susceptibility profiles. Here, we would expect to detect a delayed or reduced growth indicating only a partial activity of certain antibiotics. However, our method would not be able to distinguish mixed strains with similar susceptibility profiles. Nonetheless, since the main goal of our novel method is to rapidly generate early treatment options, rather than provide an exact ID of the infecting agent, we would consider the similarity in susceptibility profiles of multiple infecting microbes beneficial, as these should respond to a single treatment.

While we are convinced SLIC could represent a breakthrough in clinical microbiology diagnostics, this technology could also be an important step forward for the pharmaceutical industry. Indeed, the increased occurrence of antibiotic resistance in our environment has launched an important effort in the pharma sector to find new antibiotics against multi-resistant strains. The sensitivity of SLIC could accelerate this important work by enabling the rapid screening of drug efficacy within minutes. One avenue to curtail antibiotic resistance is the use of bacteriophages, a technique that has been known and used for decades but that has struggled to be widely adopted [26]. Phages are natural bacteria-targeting viruses that are widely spread wherever bacteria are found. However, phages are extremely specific and a given phage can only dock onto a specific bacterial strain before injecting its genome into the cell. This means that the lytic activity of a broad library of phages will probably need to be screened on a case-to-case basis. To allow broad clinical use, it will therefore be essential to possess rapid and sensitive activity readouts, such as SLIC.

The raw data collected from SLIC can easily analyze the kinetics of bacterial growth, using simple mathematical equations (e.g., AUCs, curve slope). When comparing the growth of a non-inhibited control to the drug-treated conditions, one can quickly determine whether a bacterial strain is sensitive or resistant to a specific drug. These calculations could be easily automated and run live through software to be developed, giving a diagnostic facility or a high-throughput screening platform real-time information on the infectious status of a patient or the activity of new chemical agents. This is at least the direction towards which we hope SLIC technology will develop.

Together, the present study delineates a strategy to quickly detect an infection and provide a clinically relevant antibiotic susceptibility profile for a variety of bacterial species. While more work will be necessary to expand our testing in primary samples and to increase the throughput of SLIC, our present data further motivates the use of SLIC for clinical microbiology diagnostics or even for the development of new antimicrobial treatments in the pharmaceutical industry.

## 4. Materials and Methods

### 4.1. Reagents

Bacteria were cultivated in Muller-Hinton Broth (Carl Roth GmbH, Karlsruhe, Germany) and prepared following the manufacturer’s recommendations. For clonal isolation, bacteria were grown on Tryptone Soya Agar (TSA) containing sheep blood (Fisher Scientific GmbH, Schwerte, Germany). Antibiotics ampicillin, cefoxitin, rifampicin, vancomycin (Carl Roth GmbH, Karlsruhe, Germany), ciprofloxacin (VWR, Darmstadt, Germany), ceftazidime, fosfomycin, meropenem, piperacillin, sulbactam, and tazobactam (Biomol GmbH, Hamburg, Germany) were purchased as powder and sterile stock solutions (12.5 mg/mL) were prepared using water (except for rifampicin which was dissolved in DMSO), aliquoted and stored at −20 °C. Amphotericin B was purchased as suspension (100 mg/mL, Dermapharm AG, Grünwald, Germany), aliquoted, and stored at −20 °C. Piperacillin/tazobactam and ampicillin/sulbactam combinations were prepared as 8:1 and 2:1 mixes, respectively. Bacteria lab strains were *S. aureus* ATCC29213 and *E. coli* WK6.

### 4.2. Patient Samples

The study was authorized by the institutional Ethics Committee of the University Hospital Bonn (ID: 110/23-EP, date: 17 July 2023). The study included any patient over 18 years old with periprosthetic joint infection seeking treatment in the Clinic for Orthopedics and Trauma Surgery of the University Hospital Bonn (UKB) between August 2023 and November 2024. Following signed consent from the patient, collected synovial fluid or tissue samples (joint capsule and membrane tissue in direct contact with prosthesis) were extracted peri-operatively and did not impede the collection of appropriate samples for diagnostic purposes. The results of our experiments were of a purely academic nature. Treatment decisions were not affected by our study and were only made based on the results from the microbiology diagnostics department.

To test our method on more clinically relevant microbes, we isolated bacterial strains from eight patients infected with commonly diagnosed bacterial strains. Tissue samples were delivered from the operation room (OR) in sterile plastic tubes containing metal beads and 5 mL sterile 0.9% NaCl solution. All following procedures were carried out in sterile conditions. Tubes containing tissue samples were vortexed for 1 min at 3000 rpm to detach the bacteria from the tissue. Samples were then transferred into a 40 µm strainer placed on a 50 mL tube. Filtrate was retained and 1 mL MHB medium as well as Li-Heparin (100 U/mL) were added. Samples were centrifuged at 1000× *g* for 10 min and supernatants were retained for further use. Joint fluid samples were delivered in sterile syringes. Samples were transferred into a 40 µm strainer placed on a 50 mL tube. Filtrate was retained and Li-Heparin (100 U/mL) was added prior to centrifugation at 1000× *g* for 10 min. Supernatants were finally retained for further use.

### 4.3. Bacteria Culture and Patient-Derived Strain Isolation

For clonal isolation of patient-derived bacterial strains, 50 µL of infected samples were placed on a TSA sheep blood agar plate and spread in 3 steps, then cultured at 37 °C for 16–24 h (depending on the growth rate). Clones were then harvested and further cultured in 7 mL MHB medium at 30 °C for 16 h, with constant rocking. Glycerol stocks were prepared from the overnight culture by mixing 600 µL culture with 400 µL sterile 50% glycerol and stored at −80 °C. The remaining culture was used for experiments. Overnight cultures were prepared freshly prior to experiments.

### 4.4. Identification of Patient-Derived Bacterial Strains

To identify the bacterial strains isolated from the patient samples, 1 mL of the overnight culture was centrifuged at 5000× *g* for 10 min. Supernatants were discarded and the pellet was washed once with 1 mL of PBS. Following a second centrifugation, the washing solution was discarded and pellets were stored dry at −80 °C. To extract the genomic DNA, pellets were thawed on ice and resuspended in 180 µL enzymatic lysis buffer (20 mM Tris-HCl pH 8.0, 2 mM EDTA, 1.2% Triton X-100, 20 mg/mL lysozyme) and incubated at 37 °C for 1 h. Proteinase K and buffer AL (DNeasy Blood & Tissue DNA kit, Qiagen, Hilden, Germany) were added and samples were incubated at 56 °C overnight. Genomic DNA was further isolated using the DNeasy kit following the manufacturer’s instructions. The ribosomal 16S genomic sequence was amplified using conventional PCR followed by electrophoresis on a 1.5% agarose gel containing PeqGreen RNA/DNA dye (PeqLab Biotechnologie GmbH, Erlangen, Germany) and imaged using a ChemiDoc MP scanner (BioRad, Feldkirchen, Germany). PCR products were purified from the gel using a Purelink Quick Gel Extraction kit (Thermo Fisher Scientific, Darmstadt, Germany) following the manufacturer’s instructions. Finally, samples were sent for Sanger sequencing (Eurofins Genomics, Ebersberg, Germany). Sequences were aligned to the NCBI S16 rRNA/ITS database using BLASTn.

### 4.5. Scattered Light Integrated Collector (SLIC)

The instrument sample chamber as well as all growth media were pre-heated to 37 °C. Pre-warmed medium was added to each cuvette (BRAND GmbH + Co KG, Wertheim am Main, Germany) together with antibiotics diluted to the desired final concentration as well as overnight bacteria cultures (diluted 1:100). The culture was homogenized, paying attention to avoid the generation of bubbles. The final volume in the cuvettes was 1.5 mL. Growing cultures were transferred directly into the sample chamber. The instrument was set to record the total laser light scatter output every second for 90 min.

### 4.6. Data Analysis and Statistics

Data generated using SLIC was analyzed using RStudio. Data from each growth curve was normalized to its baseline (i.e., the detected value of the fourth data point—4 s). AUCs were calculated using the trapezoid method of the AUC package (version 0.3.2), starting quantification from 5 min to 90 min. Normalized AUCs (norm. AUC) were calculated by dividing the test AUC by the uninhibited control AUC. The normalized curve slopes (norm. Slope) were defined by calculating the average slope of each condition (average of diff(y)/diff(t) for every data point, where y is the detected scatter output in mV and t is the time in min) and normalizing to the non-inhibited condition. Experiments were repeated at least 3 times and data are represented as average ± SEM of 3 independent experiments. Statistical analysis was performed using GraphPad Prism with either One-way ANOVA (Dunnet multiple comparisons) or Two-way ANOVA (Tukey multiple comparisons) as specified in the figure legend. Data visualization was prepared using GraphPad Prism version 10.4.1.

## Figures and Tables

**Table 1:** Antibiotic panel design. Panels of five antibiotics are summarized including their class, functional target, and the information on the microbe identity brought by susceptibility or lack of susceptibility of a tested microbe to the specified antimicrobial agent. MICs as determined in Figure 3 are indicated (µg/mL).

Antibiotic	Class	Functional Target	Information	MIC (µg/mL)
**Panel 1**				
1) Amphotericin B	Polyene	Increases cell wall permeability	Fungal	3.13
2) Ciprofloxacin	Fluoroquinolone	Gyrase inhibition	MRGN classification, Anaerobic and atypical bacteria	12.5
3) Piperacillin/ Tazobactam	Penicillin/β-lactamase inhibitor	Cell wall synthesis	MRGN classification, Pseudomonas and Enterobacter	25/3.13
4) Vancomycin	Glycopeptide	Cell wall synthesis	Gram behavior	3.13
5) Meropenem	Carbapenem	Cell wall synthesis	MRGN classification, Pseudomonas and Enterobacter	0.76
**Panel 2**				
1) Ampicillin/ Sulbactam	Penicillin/β-lactamase inhibitor	Cell wall synthesis	Antibiotic de-escalation possible?	12.5/6.25
2) Rifampicin	Ansamycin	Bacterial RNA-polymerase inhibition	Biofilm-forming bacteria	6.25
3) Fosfomycin	Phosphonic	Cell wall synthesis	Biofilm-forming bacteria	25
4) Ceftazidim	Cephalosporin (3rd gen.)	Cell wall synthesis	MRGN classification, Pseudomonas	25
5) Cefoxitin	Cephalosporin (2nd gen.)	Cell wall synthesis	MRSA classification	25

## Data Availability

The original contributions presented in the study are included in the article/Supplementary Materials. Further inquiries can be directed to the corresponding author.

## Supplementary Materials

The following supporting information can be downloaded at: https://www.mdpi.com/article/10.3390/ijms26041553/s1.
