# Supplementary material for: Novel Method for the Rapid Establishment of Antibiotic Susceptibility Profiles in Bacterial Strains Linked to Musculoskeletal Infections Using Scattered Light Integrated Collector Technology
Source: Int J Mol Sci. 2025 Feb 12;26(4):1553. doi: 10.3390/ijms26041553 (PMC11855405; doi:10.3390/ijms26041553)
Supplement: Supplementary file 1 [file ijms-26-01553-s001.zip › ijms-3436053-supplementary.pdf]

SUPPLEMENTARY MATERIAL

Figure S1

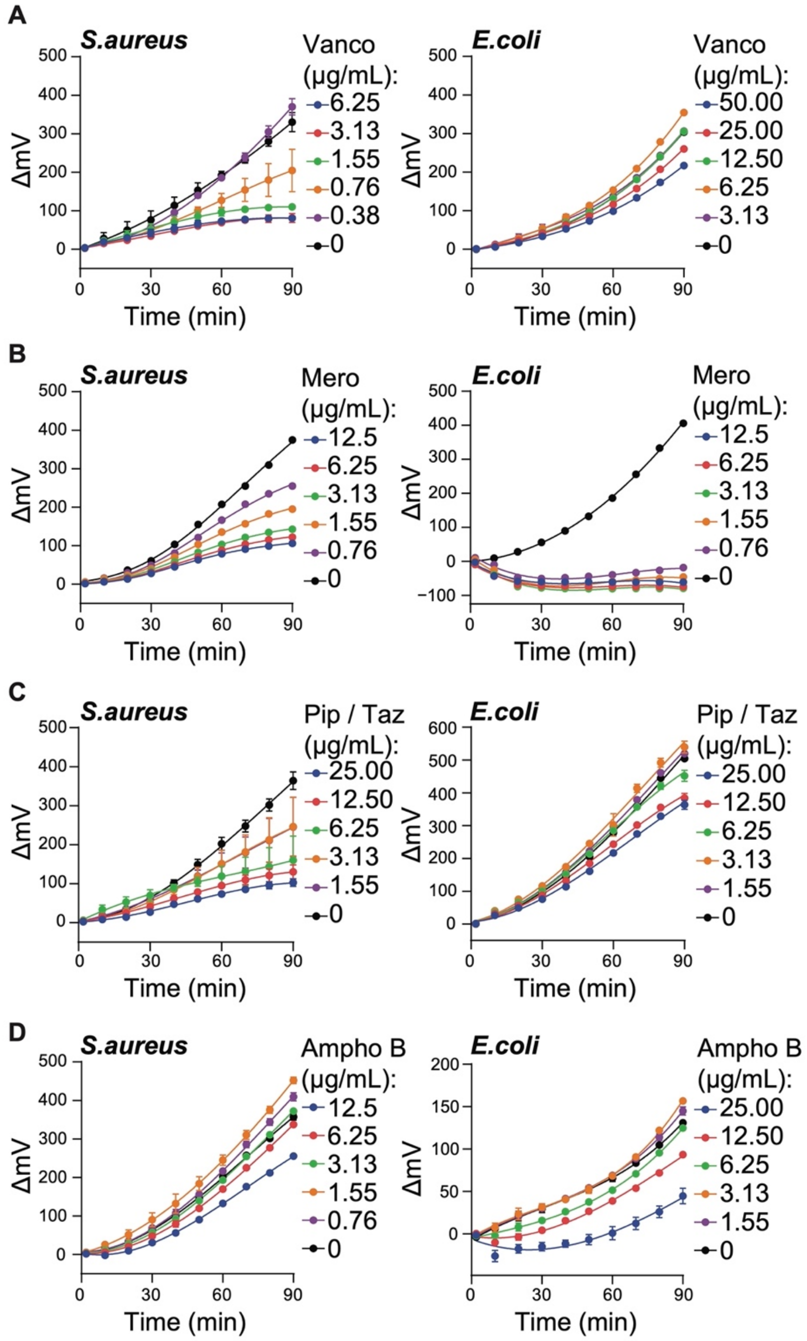

Figure S1. Establishment of MICs for antibiotic Panel 1. Lab strains of *S. aureus* (ATCC 29213) and *E. coli* (WK6) were grown at 30°C for 16h. Following a 100-fold dilution, and treatment with the indicated doses of antibiotics, cell growth was monitored in real-time for 90 min inside the SLIC chamber set at 37°C. Antibiotics titrated were Vancomycin (A), Meropenem (B), a 1-to-1 combination of Piperacillin and Tazobactam (C) or Amphotericin B (D). Data for the titration of Ciprofloxacin is shown in Figure 1A. Data represents the normalized light refraction (ΔmV) as measured over time for the growth of *S. aureus* (left panels) or *E. coli* (right panels). Data points corresponds to the average of the 60 measurements (1/sec) taken every 10th minute. Data is expressed as average ± SEM of 3 independent experiments. Growth curves were used to calculate AUCs as displayed in Figure 3.

Figure S2

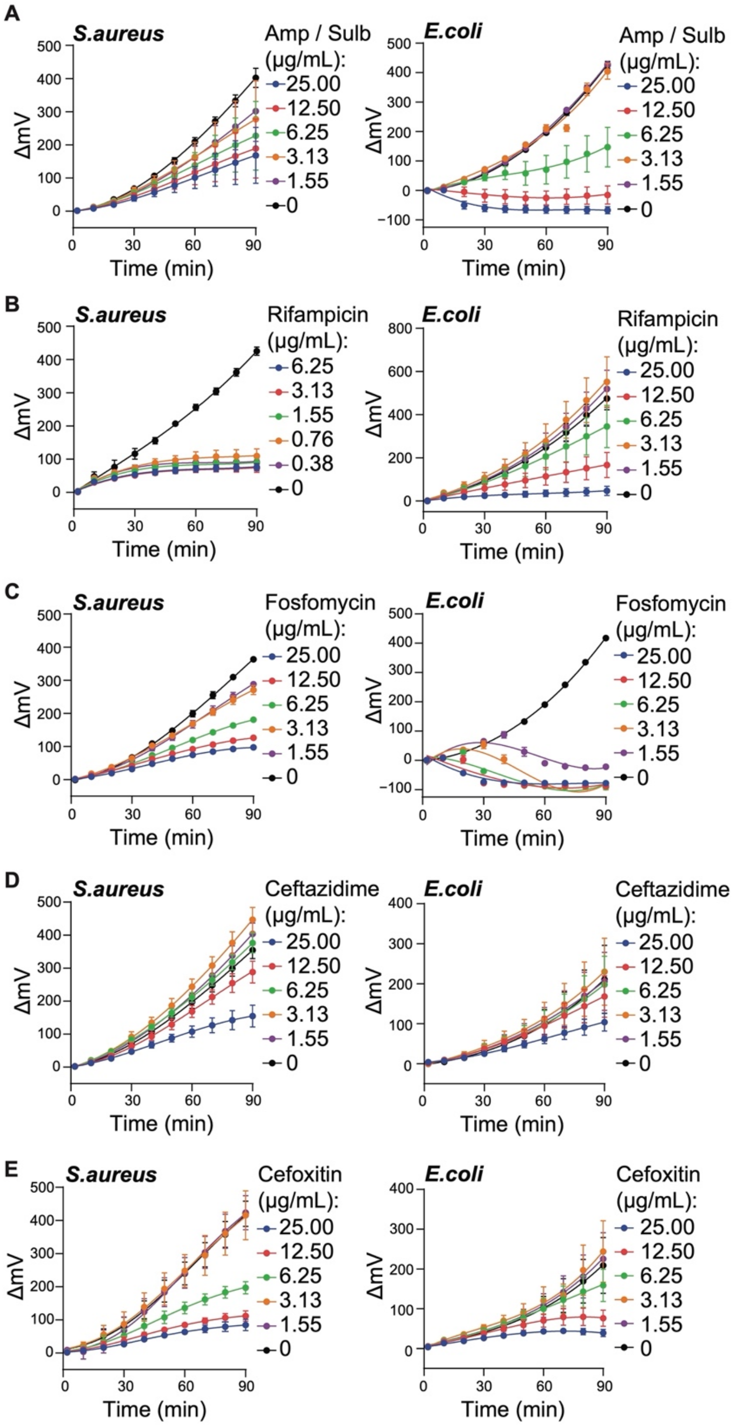

Figure S2. Establishment of MICs for antibiotic Panel 2. Lab strains of *S. aureus* (ATCC 29213) and *E. coli* (WK6) were grown at 30°C for 16h. Following a 100-fold dilution, and treatment with the indicated doses of antibiotics, cell growth was monitored in real-time for 90 min inside the SLIC chamber set at 37°C. Antibiotics titrated were a 1-to-1 combination of Ampicillin and Sulbactam (A), Rifampicin (B), Fosfomycin (C), Ceftazidim (D), or Cefoxitin (E). Data represents the normalized light refraction (ΔmV) as measured over time for the growth of *S. aureus* (left panels) or *E. coli* (right panels). Data points represent the average of the 60 measurements (1/sec) taken every 10th minute. Data is expressed as average ± SEM of 3 independent experiments. Growth curves were used to calculate AUCs as displayed in Figure 3B.

Figure S3

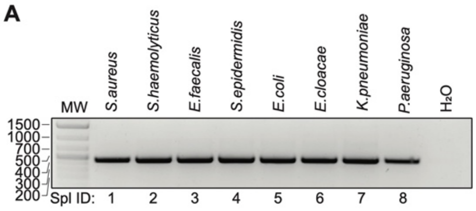

B

		BLASTn Results				
Sample ID	Expected Strain	Top 3 Species (Accession)	Total Score	Query Cover	% Identity	E Value
1	S. aureus (wild)	S. aureus (NR_037007.2)	556	100%	100%	2E-158
		S. aureus (NR_118997.2)	556	100%	100%	2E-158
		S. aureus (NR_036828.1)	556	100%	100%	2E-158
2	S. haemolyticus	S. haemolyticus (NR_113345.1)	556	100%	100%	2E-158
		S. taiwanensis (NR_181843.1)	556	100%	100%	2E-158
		S. capitis (NR_113348.1)	556	100%	100%	2E-158
3	E. faecalis	E. faecalis (NR_113902.1)	556	100%	100%	2E-158
		E. faecalis (NR_113901.1)	556	100%	100%	2E-158
		E. faecalis (NR_040789.1)	556	100%	100%	2E-158
4	S. epidermidis	S. epidermidis (NR_113957.1)	551	100%	99.67%	1E-156
		S. epidermidis (NR_036904.1)	551	100%	99.67%	1E-156
		S. caprae (NR_024665.1)	551	100%	99.67%	1E-156
5	E. coli (wild)	E. coli (NR_024570.1)	556	100%	100%	2E-158
		E. coli (NR_112558.1)	556	100%	100%	2E-158
		E. marmotae (NR_136472.1)	556	100%	100%	2E-158
6	E. cloacae	E. cloacae (NR_118011.1)	556	100%	100%	2E-158
		E. ludwigii (NR_042349.1)	556	100%	100%	2E-158
		L. adecarboxylata (NR_104933.1)	556	100%	100%	2E-158
7	K. pneumoniae	K. pneumoniae (NR_114507.1)	551	100%	99.67%	1E-156
		K. pneumoniae (NR_041750.1)	551	100%	99.67%	1E-156
		K. aerogenes (NR_102493.2)	551	100%	99.67%	1E-156
8	P. aeruginosa	P. aeruginosa (NR_114471.1)	556	100%	100%	2E-158
		P. aeruginosa (NR_113599.1)	556	100%	100%	2E-158
		P. aeruginosa (NR_117678.1)	556	100%	100%	2E-158

Figure S3. Validation of isolated strains by S16 SANGER sequencing. Indicated bacterial strains were isolated from infected patient samples (perioperative synovial fluid or tissue) by culture on solid blood agar. Bacterial clones were selected, cultured at 30°C for 16h. Cells were pelleted, lysed and genomic DNA was isolated. S16 ribosomal genomic sequence was amplified by PCR, followed by electrophoresis of the PCR products in an agarose gel (A). PCR products were finally gel extracted and sequenced by SANGER sequencing technique (Eurofins). (B) Top 3 hits from BLASTn alignment of SANGER sequences with the NCBI S16 rRNA/ITS databases. Data is representative of 1 out of at least 2 independent experiments.

Figure S4

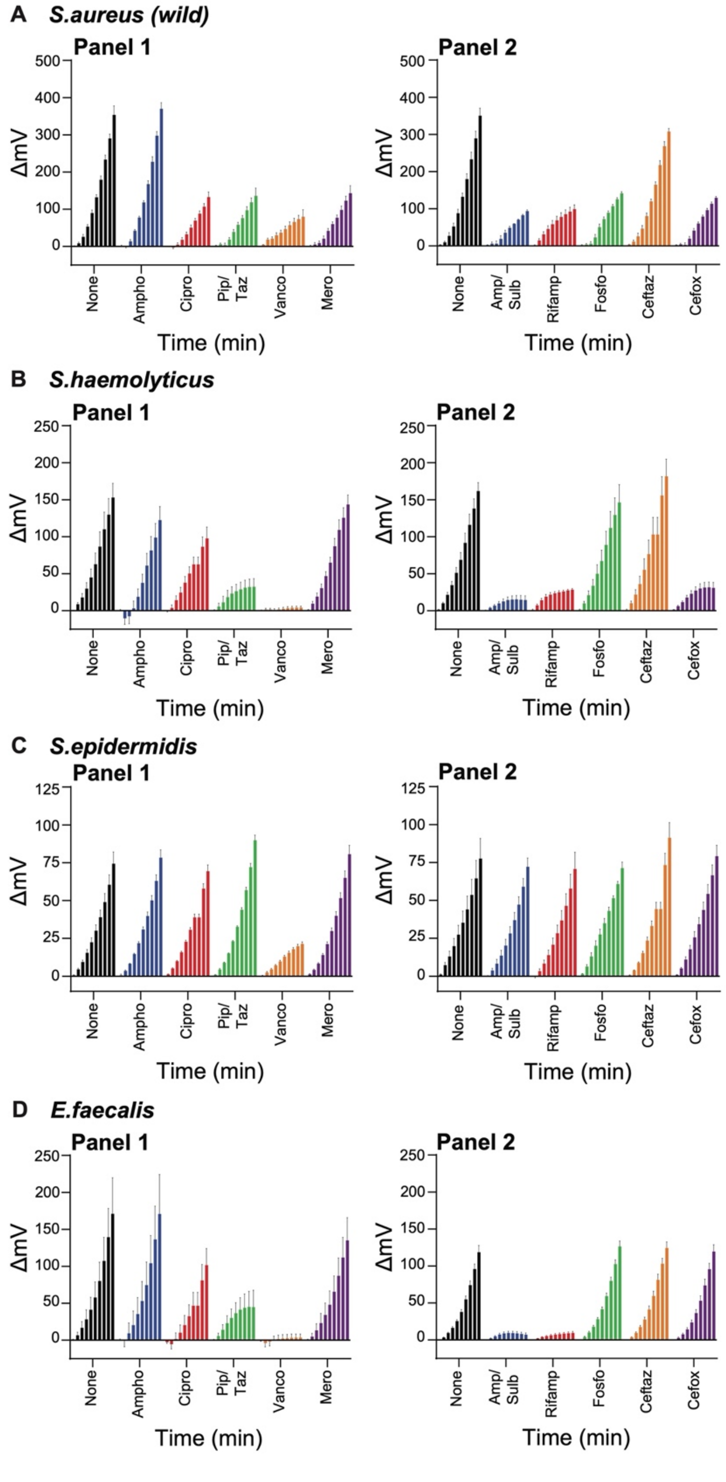

Figure S4. Growth profiles of clinically relevant gram-positive bacterial strains. Indicated bacterial strains were isolated from infected patient samples (perioperative synovial fluid or tissue) and identified using PCR and SANGER sequencing of S16 ribosomal sequence (see Figure S3). Bacteria were grown at 30°C for 16h. Following a 100-fold dilution, and treatment with indicated antibiotics at the pre-established MIC (see Table 1), cell growth was monitored in real-time for 90 min inside the SLIC chamber set at 37°C. Data represents the normalized light refraction (ΔmV) as measured over time for the growth of a wild strain of *S. aureus* (A), *S. haemolyticus* (B), *S. eperdimidis* (C), and *E. faecalis* (D). Each data point represents the average of the 60 measurements (1/sec) taken every 10th minute. Data is expressed as average ± SEM of 3 independent experiments.

Figure S5

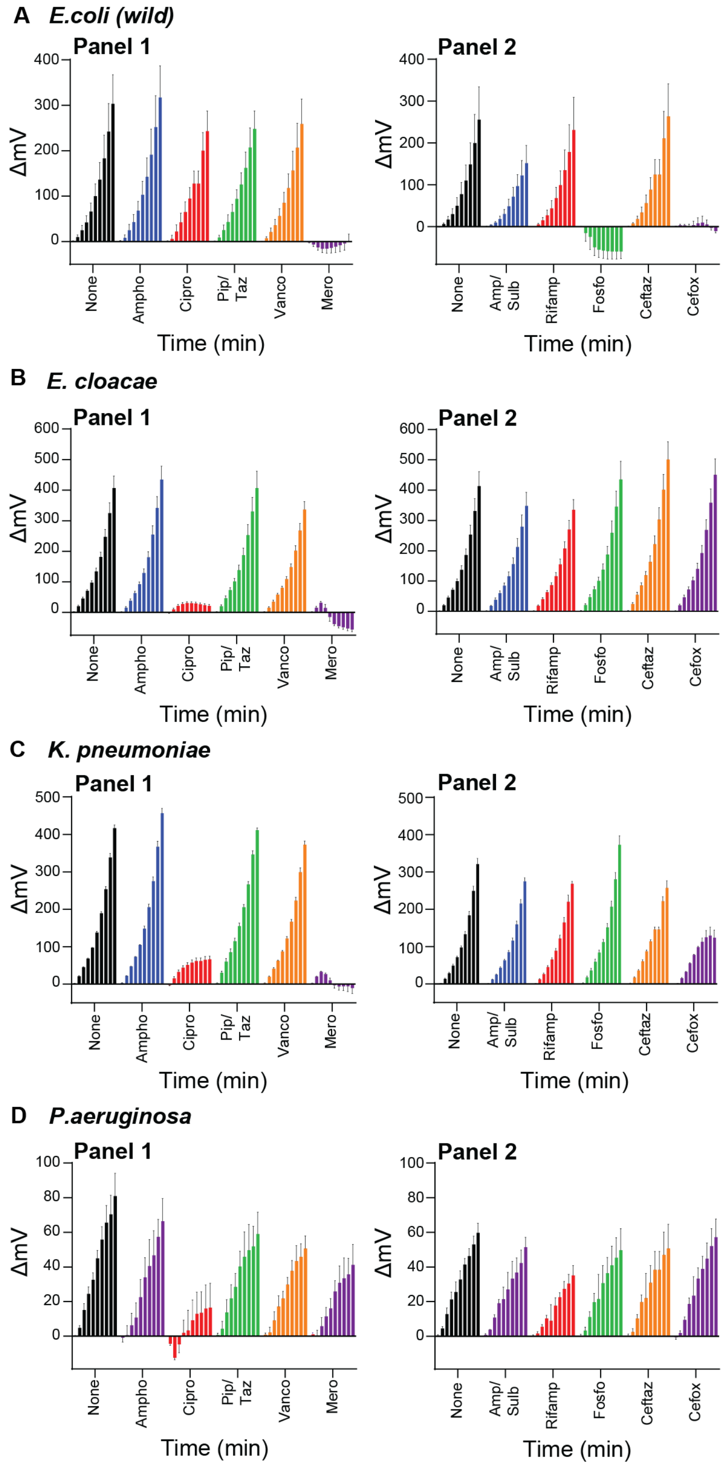

Figure S5. Growth profiles of clinically relevant gram-negative bacterial strains. Indicated bacterial strains were isolated from infected patient samples (perioperative synovial fluid or tissue) and identified using PCR and SANGER sequencing of S16 ribosomal sequence (see Figure S3). Bacteria were grown at 30°C for 16h. Following a 100-fold dilution, and treatment with indicated antibiotics at the pre-established MIC (see Table 1), cell growth was monitored in real-time for 90 min inside the SLIC chamber set at 37°C. Data represents the normalized light refraction (ΔmV) as measured over time for the growth of a wild strain of *E. coli* (A), *E. cloacae* (B), *K. pneumoniae* (C), and *P. aeruginosa* (D). Each data point represents the average of the 60 measurements (1/sec) taken every 10th minute. Data is expressed as average ± SEM of 3 independent experiments.
